# Therapeutic anticoagulation complications in the elderly: a case report

**DOI:** 10.1186/s12877-022-02781-6

**Published:** 2022-02-05

**Authors:** Marcel Niemann, Karl F. Braun, Sufian S. Ahmad, Christian Eder, Ulrich Stöckle, Frank Graef

**Affiliations:** 1 Charité - Universitätsmedizin Berlin, Center for Musculoskeletal Surgery, corporate member of Freie Universität Berlin, Humboldt-Universität zu Berlin, and Berlin Institute of Health, Augustenburger Platz 1, 13353 Berlin, Germany; 2 Department of Trauma Surgery, University Hospital Rechts der Isar, Technical University of Munich, Ismaninger Str. 22, 81675 Munich, Germany

**Keywords:** Geriatric medicine, Anticoagulation complication, Bleeding complication, Hospitalist, Case report

## Abstract

**Background:**

The demographic transition leads to a continuously growing number of elderly patients who receive therapeutic anticoagulation by reason of several comorbidities. Though therapeutic anticoagulation may reduce the number of embolic complications in these patients, major complications such as bleeding complications need to be kept in mind when considering such therapy. However, evidence regarding the choice of anticoagulation agents in chronic kidney disease patients of higher age is limited. In this report, a guideline-based anticoagulation treatment which led to a fulminant atraumatic bleeding complication is discussed.

**Case presentation:**

We present the case of an 85-year-old female stage V chronic kidney disease patient who suffered from a diffuse arterial, subcutaneous bleeding in her lower left leg due a therapeutic anticoagulation using low molecular weight heparin (LMWH). Anticoagulation was started in accordance with general recommendations for patients with atrial fibrillation, and the dosage was adapted for the patient’s renal function. Nevertheless, the above-mentioned complication occurred, and the bleeding led to a hemorrhagic shock and an acute kidney injury on top of a chronic kidney disease. The hematoma required surgical evacuation and local coagulation in the operating room. In the further course, the patient underwent additional four surgical interventions due to a superinfected skin necrosis, including skin grafting. Furthermore, the patient needed continuous renal replacement therapy, as well as intensive care unit treatment, for a total of 47 days followed by 36 days of geriatric rehabilitation. Afterwards, she was discharged from the hospital to her previous nursing home.

**Discussion and conclusions:**

Although therapeutic anticoagulation may sufficiently protect patients at cardiovascular risk, major complications such as bleeding complications may occur at any time. Therefore, physicians need to regularly re-evaluate any prior indication for therapeutic anticoagulation. With this case report, we hope to draw attention to the cohort of geriatric patients and the need for more and well differentiated study settings to preferably prevent any potentially avoidable complications.

## Background

Due to the demographic transition, the number of elderly patients who receive long-term therapeutic anticoagulation keeps increasing [1, 2]. The prevalence of atrial fibrillation as a cause of therapeutic anticoagulation is expected to continuously rise [3]. Although therapeutic anticoagulation might reduce the number of embolic complications of patients at cardiovascular risk [4], clinicians need to keep in mind that therapeutic anticoagulation increases the risk of major bleeding complications needing operative treatment [5]. Therefore, scores like the CHA2DS2-VASc-Score [6] may help clinicians to weigh up the benefits and risks of therapeutic anticoagulation of their patients. However, even if these scoring systems were adequately applied and medication dosages were chosen as recommended for an adequate indication, bleeding complications may not be avoided in every patient case. Especially in chronic kidney disease patients of higher age, data on the anticoagulation substance to choose is rare and patients are mostly not adequately represented in study designs. To discuss this as well as potential future management improvements, we will present the case of a geriatric patient with chronic kidney disease stage V and a therapeutic anticoagulation who suffered from a major bleeding complication.

## Case presentation

We present the case of an 85-year-old female patient with a weight of 88 kg and a height of 165 cm who was admitted to our emergency department following a rapidly progressing atraumatic swelling of her lower left leg. One month prior to this event, a renal function monitored therapeutic anticoagulation with Enoxaparin sodium 80 mg/0.8 ml (Clexane®, ﻿Sanofi-Aventis Deutschland GmbH, Frankfurt am Main, Germany) was initiated due to persistent atrial fibrillation and chronic kidney disease (estimated glomerular filtration rate (eGFR) < 15 ml/min at the time of therapeutic anticoagulation initiation). Unfortunately, baseline CHA2DS2-VASc- and HAS-BLED scores were not provided at the initial hospital admission.

Besides the chronic kidney disease (anti-nuclear antibodies negative, anti-neutrophil cytoplasmic antibodies negative, and immunofixation negative), the patient suffered from a secondary hyperparathyroidism, persistent atrial fibrillation, hypertension, and coronary artery disease.

**Fig. 1:**
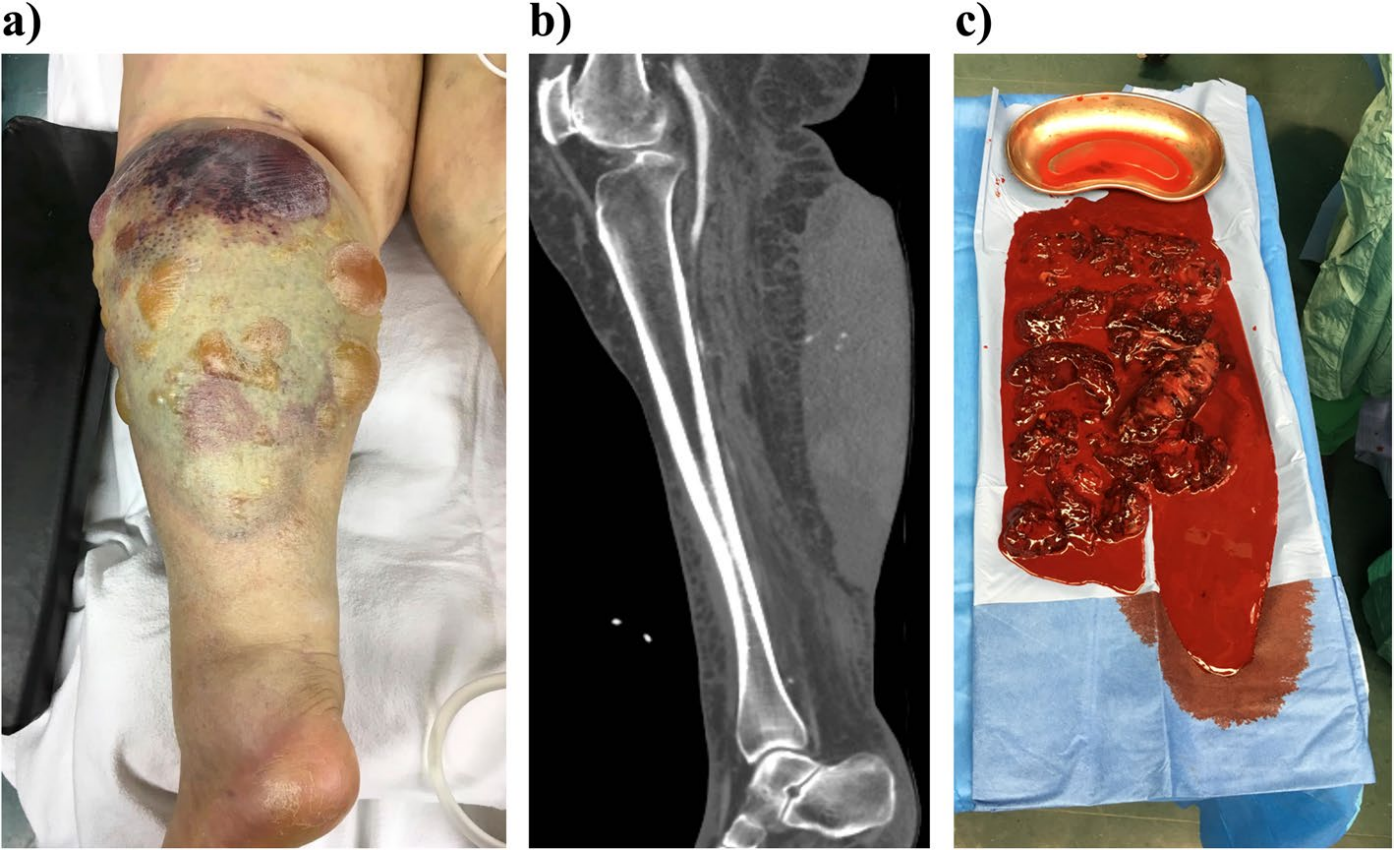
Case presentation of a complication following anticoagulation. **a**) Depicts the clinical presentation of the patient in the emergency department. **b**) exemplary displays some arterial bleeding locations. **c**) depicts the amount of operatively evacuated hematoma

At the initial assessment, the patient was pale and presented with a massive swelling of her lower left leg due to a subcutaneous hematoma, which led to various epidermal blisters (Fig. 1a). Furthermore, she was hypotensive with a blood pressure of 85/60 mmHg and anemic with a hemoglobin of 5.3 g/dl. The remaining values of the complete blood count were as follows: Hematocrit 0.207 l/l [0.350-0.455], red blood cell count 2.4/pl [3.9-5.1/pl), mean corpuscular volume (MCV) 86.0 fl [80.0-101.0 fl], mean corpuscular hemoglobin (MCH) 28.6 pg [27.0-34.0 pg], mean corpuscular hemoglobin concentration (MCHC) 33.3 g/dl [31.5-36.0], white blood cell count 6.32/nl [3.90-10.50/nl], platelet count 220/nl [150-370]. Furthermore, the serum creatinine was 2.25 mg/dl [0.50-0.90 mg/dl] and the eGFR was 19 ml/min. The antifactor Xa assay for low molecular weight heparin (LMWH) revealed a score of 0.24 U/ml (reference range for therapeutic anticoagulation: 0.8-1.5 U/ml), therefore an overdose was out of the question. Two red cell concentrates were transfused immediately and a computed tomography angiography was performed which demonstrated multiple bleedings from small arteries (Fig. 1b). Decision was made to evacuate the hematoma and surgically control the bleeding in an effort to reduce the risk of expected cutaneous necrosis as a result of the elevated tissue pressure. During surgery, around two liters of partially clotted blood were removed from the patient’s leg (Fig. 1c). Afterwards, the patient was transferred to the intensive care unit (ICU) where she was treated by continuous renal replacement therapy due to the acute on chronic renal failure which was assumed to be a result of the hypovolemic shock. The therapeutic anticoagulation was stopped, and the patient received unfractionated heparin in a prophylactic dosage.

Thirteen days after hospital admission and the aforementioned operative treatment, the patient developed a superficial skin necrosis which became infected and required radical necrosectomy and vacuum therapy. Following repeated vacuum therapy dressing changes, the skin defect could finally be covered by an autologous split-thickness skin graft 4 weeks after the admission to the hospital.

In total, the patient needed an ICU treatment for 47 days with a continuous and, later on, intermittent dialysis followed by 36 days of geriatric rehabilitation. Afterwards, the patient was discharged to her previous nursing home.

## Discussion and conclusion

In the presented case, the CHA2DS2-VASc-Score of the patient was five (one point for high blood pressure, two points for age ≥ 75 years, one point for coronary heart disease, and one point for female sex) resulting in a thromboembolic risk rate of 3.2% (95% CI, 0.7–9.0%) per year [6]. Therefore, a therapeutic anticoagulation had been initiated and previous physicians had decided against the primarily indicated coumarins and the directly acting oral anticoagulants (DOACs). DOACs had been excluded due to the chronic kidney disease with an estimated glomerular filtration rate < 15 ml/min and coumarins had been excluded because the inevitably necessary laboratory monitoring of the patient could not be assured [7–9]. LMWH had been chosen as anticoagulant and had been adapted for the patient’s renal function according to the manufacturer’s specification [10]. This adaption had been made in accordance with previous data aiming to avoid an antifactory Xa accumulation resulting in a higher risk of bleeding complications [11]. Accordingly, the therapeutic anticoagulation had been adequately established in accordance with the official recommendations. This was confirmed by an antifactor Xa assay for LMWH at the hospital admission that scored below the reference range for a therapeutic anticoagulation with LMWH. This matches previous study’s data on the predictability of antifactory Xa values following renal function adapted Enoxaparin injections [12]. Basically, the LMWH dosage should have been increased in order to adequately reduce the thromboembolic risk. Following the aforementioned atraumatic bleeding of the lower extremity, however, the anticoagulation had to be stopped in accordance with a HAS-BLED score of five (one point for high blood pressure, one point for abnormal renal function, one point for bleeding history, one point for age > 65 years, and one point for the intake of non-steroidal anti-inflammatory drugs) resulting in a bleeding risk > 8.70 bleeds per 100 patient-years [13].

Physicians need to keep in mind that a therapeutic anticoagulation entails even more challenging problems within elderly patients, who commonly suffer from other diseases such as chronic kidney disease. While the global prevalence of chronic kidney disease was 9.1% in the global population [14], the prevalence increases up to 48% in patients older than 70 years [15]. Especially in chronic kidney disease stage four and five patients, data about anticoagulants is limited [16]. As commonly recommended in Europe in this specific patient cohort, clinicians are encouraged to prefer coumarins over DOACs [17]. However, according to previous authors reviewing the existing evidence, coumarins, such as warfarin, entail the risk of over-anticoagulation in renal failure patients despite being hepatically metabolized [9, 18]. Due to the renal excretion, LMWH may as well accumulate in these patients [9, 11, 12, 16, 18, 19]. Therefore, several authors recommended a dosage reduction and regularly scheduled laboratory follow-ups for both agents when administered in chronic kidney disease patients, for example a dose reduction to 150 U / kg / day of enoxaparin [12] or a dosage according to the manufacturers’ recommendations [11, 16]. Though, the risk of bleeding complications may still be elevated and, therefore, these agents may not be recommended in every chronic kidney disease patient [11]. Furthermore, in a huge proportion of chronic kidney disease patients, some LMWH available are not approved by the manufacturers, either, thereby limiting the variety of anticoagulants even more. This needs to be considered, especially in geriatric patient cohorts. DOACs are partially renally eliminated with the lowest renal dependency for apixaban. In accordance with the Kidney Disease: Improving Global Outcomes controversies conference and several other previous authors, the dose reduced usage of 2.5 mg apixaban twice per day may be recommended in chronic kidney disease patients [9, 19–21]. However, this recommendation is not globally valid. In Europe, for example, apixaban is contraindicated in patients with an eGFR < 15 ml/min.

The presented case entails both strengths and limitations. This scenario was observed in Europe where different pharmacological recommendations are valid, as discussed above, when compared to other countries. However, this patient case stresses the importance of clear and globally available pharmacological recommendations, especially in specific subpopulation such as geriatric patient. This patient group is continuously growing which makes clear recommendations inevitable.

In summary, choosing the anticoagulation agent potentially best matching each patient depends on several factors, especially in the geriatric cohort. Physicians need to be up to date on recent data concerning the anticoagulation in chronic kidney disease patients as these data are, right now, lacking in some countries. Although LMWH have been recommended by several authorities and used in the clinical setting for a relevant time by now, newer agents such as DOACs entail several benefits in the geriatric cohort including a less renal depending elimination and antidots available in several countries. However, the definite agent choice remains an individual decision in these patients and, therefore, general recommendations towards a therapeutic anticoagulation in chronic kidney disease patients cannot be made up until now. Furthermore, even if there is a relevant benefit of therapeutic anticoagulation in several patients for various diagnoses, clinicians should always be aware of acute complications, even in cases of long-term therapies. They should re-evaluate the indication for therapeutic anticoagulation frequently in the follow-up of their patients, especially when hospitalized. Furthermore, the decision towards a therapeutic anticoagulation in chronic kidney disease patients should only been made in accordance with a hematologist involved in the patient case. Any decision should be based on risk stratification scores such as the HAS-BLED and the CHA2DS2-VASc-Score, but each score’s limitations need to be kept in mind [22]. The lack of homogenous study results and sufficient evidence was observed at the latest KDIGO Controversies Conference, as well [22]. The authors discussed several primary and secondary stroke prevention strategies in chronic kidney disease patients. They concluded, that anticoagulation therapy in chronic kidney disease may be generally recommended, but, especially, in patients with an eGFR ≤30 ml/min the specific choice of agent should be individually discussed.

## Data Availability

Not applicable.

## Abbreviations

DOAC: Directly acting oral anticoagulant
GFR: Glomerular filtration rate
ICU: Intensive care unit
LMWH: Low molecular weight heparin
